# Development of High-Precision NO_2_ Gas Sensor Based on Non-Dispersive Infrared Technology

**DOI:** 10.3390/s24134146

**Published:** 2024-06-26

**Authors:** Yongmin Zhao, Congchun Zhang, Guangteng Ci, Xiaoguang Zhao, Jinguang Lv, Jingqiu Liang, Anjie Ming, Feng Wei, Changhui Mao

**Affiliations:** 1 State Key Laboratory of Advanced Materials for Smart Sensing, GRINM Group Co., Ltd., Beijing 100088, China; 2 Department of Advanced Electronic Materials, GRIMAT Engineering Institute Co., Ltd., Beijing 101407, China; 3 Department of Precision Instrument, Tsinghua University, Beijing 100084, China; 4 State Key Laboratory of Applied Optics, Changchun Institute of Optics, Fine Mechanics and Physics, Chinese Academy of Sciences, Changchun 130033, China; 5 GRINM (Guangdong) Institute for Advanced Materials and Technology, Foshan 528000, China

**Keywords:** non-dispersive infrared (NDIR), infrared pyroelectric detector, ultra-thin LiTaO_3_ layer, NO_2_ gas sensor

## Abstract

Increasing concerns about air quality due to fossil fuel combustion, especially nitrogen oxides (NO_x_) from marine and diesel engines, necessitate advanced monitoring systems due to the significant health and environmental impacts of nitrogen dioxide (NO_2_). In this study, a gas detection system based on the principle of the non-dispersive infrared (NDIR) technique is proposed. Firstly, the pyroelectric detector was developed by employing an ultra-thin LiTaO_3_ (LT) layer as the sensitive element, integrated with nanoscale carbon material prepared by wafer-level graphics technology as the infrared absorption layer. Then, the sensor was hermetically sealed using inert gas through energy storage welding technology, exhibiting a high detectivity (D*) value of 4.19 × 10^8^ cm·√Hz/W. Subsequently, a NO_2_ gas sensor was engineered based on the NDIR principle employing a Micro Electro Mechanical System (MEMS) infrared (IR) emitter, featuring a light path chamber length of 1.5 m, along with integrated signal processing and software calibration algorithms. This gas sensor was capable of detecting NO_2_ concentrations within the range of 0–500 ppm. Initial tests indicated that the gas sensor exhibited a full-scale relative error of less than 0.46%, a limit of 2.8 ppm, a linearity of −1.09%, a repeatability of 0.47% at a concentration of 500 ppm, and a stability of 2% at a concentration of 500 ppm. The developed gas sensor demonstrated significant potential for application in areas such as industrial monitoring and analytical instrumentation.

## 1. Introduction

With the increasing emissions of harmful gases into the environment during the combustion of fossil fuels for power generation and industrial production, air quality has quickly become a concern. NO_x_ is mainly generated by the exhaust emissions from ships and automotive diesel engines, with the NO_2_ concentration in the exhaust gas being about ten times that of nitric oxide (NO). NO_x_ is defined as the sum of all nitrogen oxides, usually mainly referring to the sum of the nitric oxide compounds NO and NO_2_. Both of thes nitrogen oxides have toxicity, with NO being less active, but easily oxidized into NO_2_ in the air. The latter has a strong corrosiveness and toxicity, mainly damaging the respiratory tract and human nervous system, seriously endangering human health [1,2,3]. Moreover, NO_2_ is more stable in the air and plays an important role in the formation of ozone holes. Therefore, it is necessary to develop NO_2_ gas sensors to meet various applications, including diesel vehicle emission monitoring.

There are many techniques reported in the literature for NO_2_ gas sensors, including electrochemical sensors, optical sensors, and semiconductor sensors, etc. Semiconductor gas sensors are the most exploited due to their ease of fabrication. Shendage et al. (2017) reported a conductometric NO_2_ gas sensor based on WO_3_ nanoplates and demonstrated a relatively low response value of 10 at a 100 ppm concentration and a 100 °C operating temperature with detection limit of 5 ppm [4]. Surbhi Jain et al. (2022) reported a NO_2_ gas sensor of Long-Range Surface Plasmons (LRSPs) and demonstrated a high sensitivity (0.2°/ppm) and a limit of detection of 0.38 ppm [5]. Shengbo Sang et al. reported In_2_O_3_ nanoparticles modified with GaN for NO_2_ detection and exhibited a response as high as 1070.9–1 ppm NO_2_ at a working temperature of 100 °C, as well as a low limit of detection (1 ppb) [6]. Semiconductor gas sensors have some major setbacks such as a low sensitivity and high-temperature operation, leading to more consumption of power and an inferior selectivity. The disadvantages of electrochemical sensors mainly include a short life, the need for regular maintenance, and their high dependence on environmental conditions. Consequently, it is very important to develop NO_2_ gas sensors with a high selectivity, long life, and high stability. Fei Yi et al. (2020) proposed a multiplexed NDIR gas-sensing platform consisting of a narrowband infrared detector array as a read-out and demonstrated a NO_2_ gas detection limitsof 54 ppm [7]. Shelly John Mechery et al. reported a fiber-optic-based sensor system for the detection of NO_2_ in air samples. A part per billion (ppb)-level detection was achieved with the sensor design [8]. Mel F. Hainey et al. demonstrated a dual-band metal–dielectric–metal cavity detector. NDIR NO_2_ gas sensing exhibited a 10 ppm accuracy and 1 ms response times [9]. Due to the complex composition of the emitted gases, it is a great challenge to accurately measure the NO_2_ concentrations in harsh environments. Optical methods use specific spectral information to identify gases and avoid interference from other gases. Jing Li et al. presented a new strategy based on the linear calibration-free wavelength modulation spectroscopy method to eliminate the effects of water interference by resolving overlapped harmonic signals. NO_2_ gas sensing exhibited ppm-level NO_2_ detection with a high H_2_O interference [10]. Optical sensors have always been a remarkable area of research, as they are ideal for complex environments.

**Figure 1:**
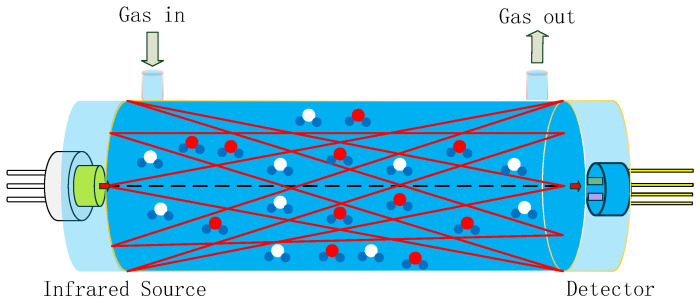
Schematic diagram of NDIR system, composed of an infrared source, optical gas chamber, and infrared detector (red and blue circles represent detecting gas molecules, white and blue circles represent other gas molecules).

Gas sensors utilizing NDIR technology are highly regarded on the market for their exceptional selectivity, rapid response time, precise accuracy, extended service life, and robust stability when compared to alternative sensor types [11,12]. A typical NDIR gas sensor system based on infrared absorption comprises an infrared (IR) emitter, optical gas chamber, IR detector, and signal-processing circuit, as shown in Figure 1 [13,14,15]. IR emitters include lasers, lamps, and MEMS IR-emitters, etc. [16]. Our previous work reported on an NDIR multi-gas sensor system for the early thermal runaway warning of automotive batteries [17]. The major drawback was the detection limit of the NDIR sensor. The detection limit is a measure of the smallest concentration which can be determined with a specified precision. The detection limit of an NDIR sensor is a function of both the voltage signal strength and signal stability. The detection limit of NDIR sensors is influenced by the intensity of the IR emitter, the design of the optical path, and the detector. In this study, NDIR NO_2_ gas sensors were developed using a non-coherent MEMS IR emitter with broad spectral coverage as the source, along with a dual-channel pyroelectric detector equipped with two optical filters for the simultaneous measurement of NO_2_ and reference signals, within a 1.5 m long white chamber. The white chamber was based on multiple reflections between spherical concave mirrors, which all had the same radius of curvature. The optical set-up featured a high light transmission, where radiation losses were caused only by absorption and scattering on the reflecting surfaces. The optical path was dependent on the adjustment of the mirrors. A long optical path of 1.5 m was implemented in a volume of 3 × 3 × 12 cm^3^. Limitations such as the accuracy, detection limit, repeatability, and stability of the sensor were overcome by improvements made to the gas chamber and pyroelectric detector.

## 2. Non-Dispersive Infrared Gas Sensor Theory

When infrared radiation passes through gas molecules with spectrum absorption characteristics, the gas molecules will absorb part of the infrared radiation. According to the Beer–Lambert law [18,19,20], the concentration of the gas can be calculated by Equation (1):

(1) $$I\left(\lambda \right)=I_{0}\left(\lambda \right)\cdot {\mathrm{e}}^{-klx}$$

where *λ* is the gas absorption wavelength, *x* is the concentration of the gas, *k* represents the correlation coefficient of gas absorption, and *l* is the optical path length between the light and the detector. *I*_0_*(λ)* and *I(λ)* are the light intensity before and after absorbing the target gas.

**Figure 2:**
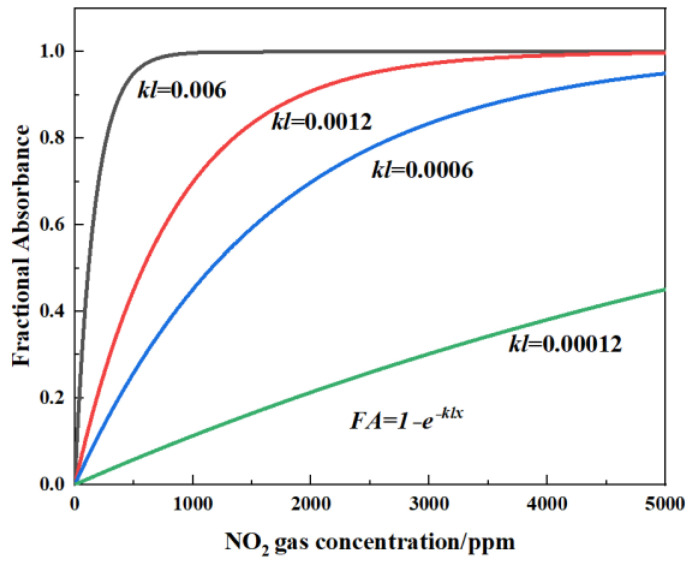
Schematic diagram of theoretical calculation of fractional absorbance (*FA*) variation with NO_x_ concentration under different light path lengths for *kl* = 0.006, 0.0012, 0.0006, and 0.00012.

The relationship between the voltage of the detector and the gas concentration can be calculated from Equation (2). *FA* is defined as the fractional absorbance of the target gas. If *k* and *l* are constant, *FA* can be plotted against *x*, as shown in Figure 2 (where *kl* = 0.006, 0.0012, 0.0006, and 0.00012). This relationship implies that long optical path length is more suited for low gas concentrations, which can also be clearly seen in Figure 2.

(2) $$FA=\frac{I_{0}-I}{I_{0}}=\frac{V_{0}-V}{V_{0}}=1-{\mathrm{e}}^{-klx}$$

where *FA* is the fractional absorbance, *V*_0_ is the voltage of the detector in N_2_ gas, and *V* is the voltage of the detector in target gas.

Due to the influence of factors such as the IR emitter and light path, practical considerations require modifications to the Beer–Lambert Law Equation (3) [21]:

(3) $$FA=\frac{V_{0}-V}{V_{0}}=SPAN\cdot \left(1-{\mathrm{e}}^{-klx^{c}}\right)$$

The *SPAN* constant depends on the range of concentration measured. The values of *kl* and *c* for a particular system are usually determined by taking a number of data points for the *FA* and the concentration of the target gas.

For a given system where the *kl* and *c* constants have been determined, the values of ZERO and SPAN can be calculated using the two-point calibration method. Firstly, low-concentration gas is injected into the gas chamber, and the relationship between voltage and concentration is given by Equation (4). Then, a high concentration of gas is passed into the gas chamber, and the relationship between the voltage and the concentration is given by Equation (5). ZERO and SPAN can be calculated by Equations (6) and (7).

(4) $$1-\frac{I_{LOW}}{I_{0}}=1-\frac{U_{LOW}}{U_{0}}=1-\frac{U_{gas-LOW}{\cdot U}_{ref-0}}{U_{ref-LOW}\cdot U_{gas-0}}=SPAN\cdot \left(1-{\mathrm{e}}^{-klx_{LOW}^{c}}\right)$$

(5) $$1-\frac{I_{CAL}}{I_{0}}=1-\frac{U_{CAL}}{U_{0}}=1-\frac{U_{gas-CAL}{\cdot U}_{ref-0}}{U_{ref-CAL}\cdot U_{gas-0}}=SPAN\cdot \left(1-{\mathrm{e}}^{-klx_{CAL}^{c}}\right)$$

(6) $$I_{0}=ZERO=\frac{U_{gas-LOW}\cdot \left({\mathrm{e}}^{-klx_{CAL}^{c}}-1\right)\cdot U_{ref-CAL}+U_{gas-CAL}\cdot {\left({1-\mathrm{e}}^{-klx_{LOW}^{c}}\right)\cdot U}_{ref-LOW}}{\left({\mathrm{e}}^{-klx_{CAL}^{c}}-{\mathrm{e}}^{-klx_{LOW}^{c}}\right)\cdot U_{ref-CAL}\cdot U_{ref-LOW}}$$

(7) $$SPAN=\frac{U_{gas-CAL}\cdot U_{ref-LOW}+U_{gas-LOW}\cdot U_{ref-CAL}}{U_{gas-LOW}\cdot \left({\mathrm{e}}^{-klx_{CAL}^{c}}-1\right)\cdot U_{ref-CAL}+U_{gas-CAL}\cdot \left(1-{\mathrm{e}}^{-klx_{LOW}^{c}}\right)\cdot U_{ref-LOW}}$$

where *I_LOW_* is the light intensity after absorbing the low-concentration gas and I_CAL_ is the light intensity after absorbing the high-concentration gas. *U_gas-LOW_* and *U_ref-LOW_* are the voltages of the reference channel after absorbing the low-concentration gas. *U_gas-CAL_* and *U_ref-CAL_* are the voltages of the gas channel after absorbing the high-concentration gas. U_ref-0_ is the voltage of reference channel before absorbing the gas, *U_gas-_*_0_ is the voltage of the gas channel before absorbing the gas. *x_LOW_* is the low-concentration gas and *x_CAL_* is the high-concentration gas.

Based on the above theory, the actual concentration of the target gas can be derived from Equations (8) and (9):

(8) $$FA=SPAN\cdot \left(1-{\mathrm{e}}^{-klx^{c}}\right)=1-\frac{U_{gas}}{U_{ref}\cdot ZERO}$$

(9) $$x={\left[\frac{ln\left(1-\frac{FA}{SPAN}\right)}{-kl}\right]}^{\frac{1}{c}}$$

where *U_gas_* is the voltage of the gas channel in an unknown gas and *U_ref_* is the voltage of the reference channel in an unknown gas.

## 3. Device Design and Fabrication

### 3.1. Design and Preparation of NDIR Gas Sensor

**Figure 3:**
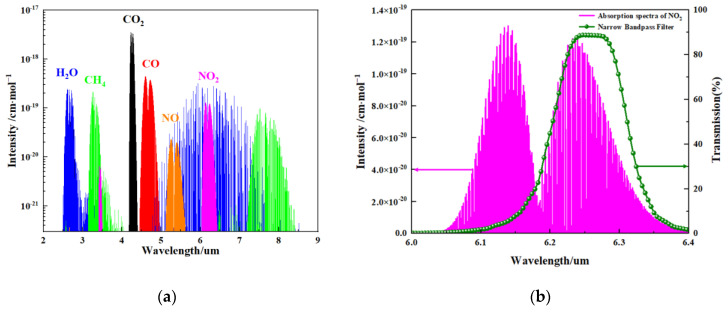
(**a**) Absorption spectra of molecules with their relative intensities. H_2_O: water; CH_4_: methane; CO_2_: carbon dioxide; CO: carbon monoxide; NO: nitric oxide; NO_2_: nitrogen dioxide; and (**b**) the absorption spectrum of NO_2_ gas and transmission spectrum of the narrow band-pass filter.

The basic principle of the NDIR gas sensor is to calculate the gas concentration by measuring the change in the infrared light absorption intensity of the target gas. NO_2_ gas molecules have an absorption peak in the mid-infrared spectral region, as shown in Figure 3a. A wavelength of 6.25 μm is chosen for the NO_2_ gas absorption in this work, as NO_2_ gas presents strong absorption peaks at a wavelength of 6.25 μm, as shown in Figure 3b. The main reason for this choice is that the absorption peak of NO_2_ has very little or negligible overlap with the absorption bands of other common gases or water vapor in the air [22,23].

The gas channel signal will decrease when infrared light passes through the NO_2_ gas. Changes in the signal and gas concentration conform to the Beer–Lambert law. Table 1 shows the main materials and dimensions of the gas sensor. Here, a commercial MEMS IR emitter (JSIR350, Micro-Hybrid Electronic GmbH, Hermsdorf, Germany) which emits a wide spectrum of infrared light is used in this system. In addition, based on the theoretical calculation above, a white cell with an optical path length of 1.5 m is selected within the target concentration range, and the infrared light source and detector are located at specific positions on both sides of the gas chamber [24].

**Figure 4:**
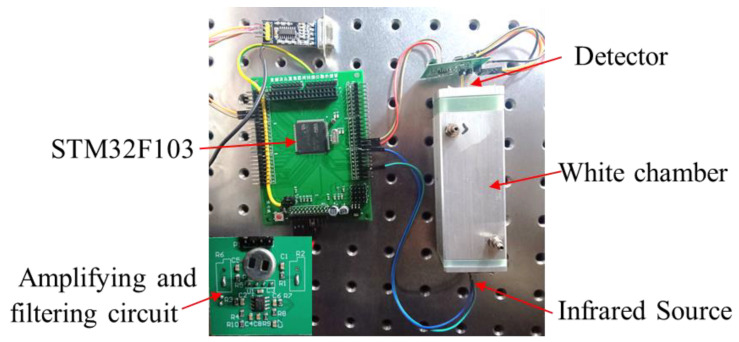
The photo of the NDIR NO_2_ sensor system developed.

**Figure 5:**
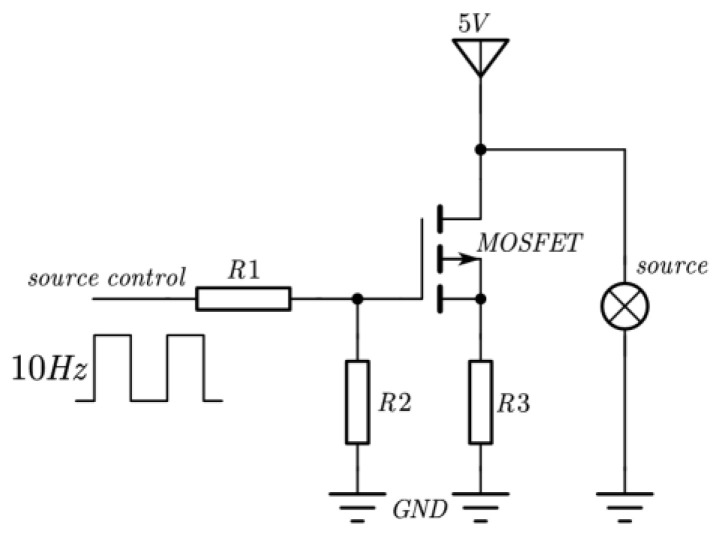
Schematic diagram of infrared source drive circuit.

**Figure 6:**
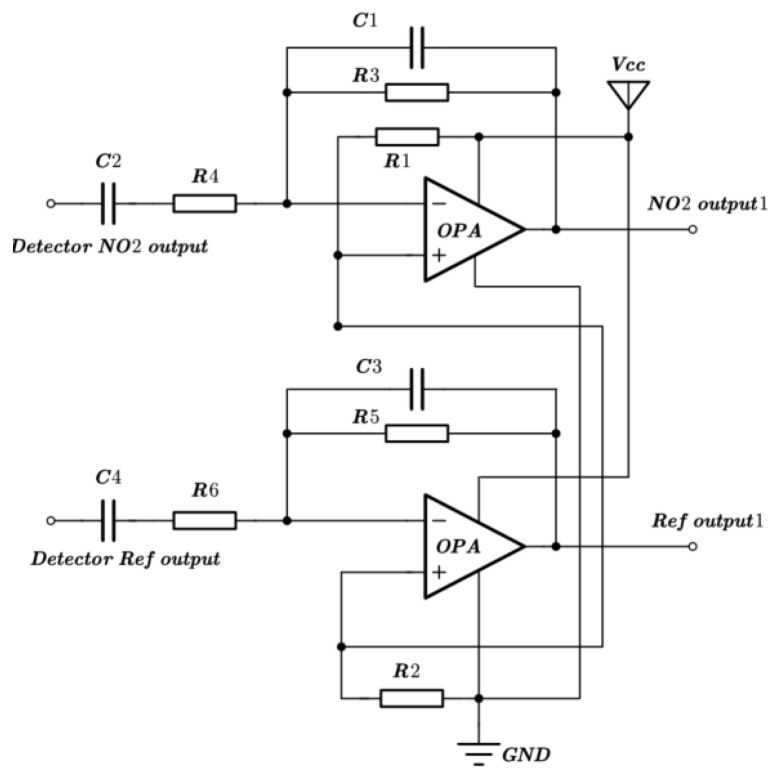
Schematic diagram of amplifying and filtering circuit.

The NO_2_ sensor system is shown in Figure 4. STM32F103 is chosen as the micro-control unit (MCU). It was used to collect and process the voltage signal from the detector to control the infrared source driver module. It can finally calculate the actual gas concentration according to the algorithm. The gas sensor system includes a 5 V–3 V voltage conversion circuit used to supply power to the MCU, amplifying and filtering the circuit [25]. A 10 Hz 50% duty cycle square wave is recommended to drive the infrared source. It is important that the frequency is generated from the MCU. The infrared source drive peak-to-peak voltage should be 5 V. The infrared source will give the maximum infrared emission and best system performance. Heat from the infrared source will also keep the temperature of the optical reflector higher than ambient. The infrared source drive current is relatively large compared to detector. The drive circuit design is required to prevent the breakthrough of the infrared source switching pulses causing voltage steps on the output waveforms. It is strongly recommended that separate voltage regulators be used for the infrared source drive and the output signal circuit. The infrared source drive current can be switched using a MOSFET. Figure 5 shows circuits for driving the infrared source. The output signal of the pyroelectric detector is very small, because infrared light becomes smaller when it passes through the air chamber. The output signal of the detector must be amplified so that the analog-to-digital converter (ADC) can detect the voltage signal. The amplifier should be mounted as close as possible to the detector to reduce the pickup of noise and other electromagnetic interference. Figure 6 shows the circuit for amplifying and filtering the detector outputs using a single 3 V supply. The circuit gain can be changed by changing the value of R3/R4 and R5/R6.

### 3.2. Design and Fabrication of Pyroelectric Detector

A pyroelectric infrared detector is the key core component of NDIR gas sensors. In order to achieve high-precision NO_2_ detection and identification, the design and processing of the developed high-performance pyroelectric infrared detector are introduced. Table 1 shows the parameters of the designed pyroelectric detector.

**Figure 7:**
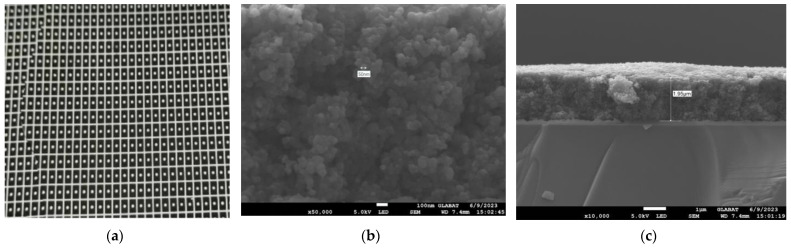
(**a**) Optical image of the wafer level carbon black absorption layer on 3 inch LT substrate; (**b**) SEM micrograph of carbon black absorption structure; and (**c**) SEM micrograph of carbon black absorption layer thickness.

**Figure 8:**
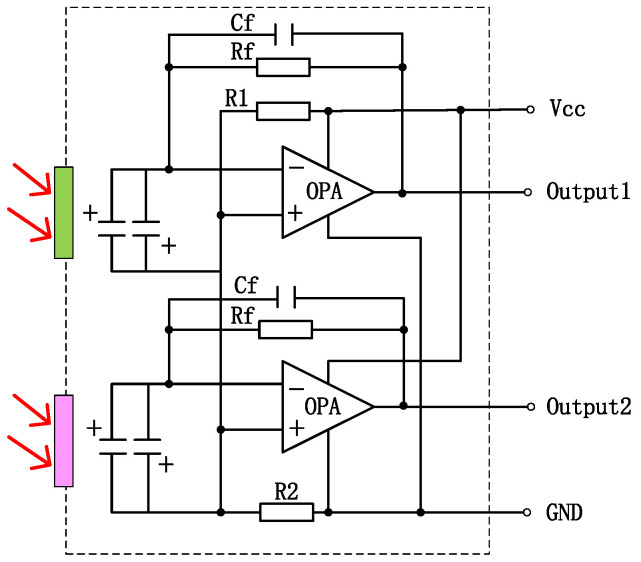
Dual-channel current-mode circuit diagram, operational amplifier to increase signal amplitude (The red arrow represents incoming infrared light).

**Figure 9:**
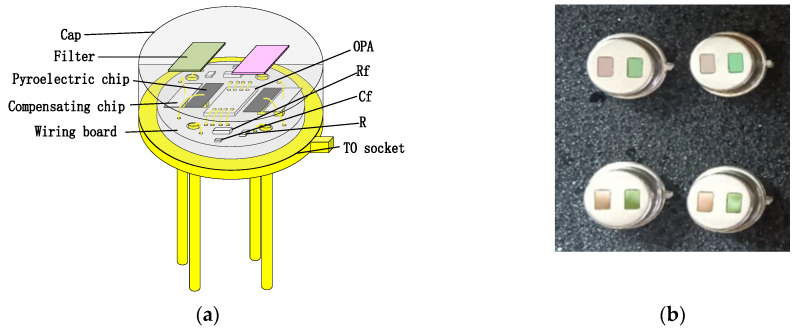
(**a**) Schematic diagram of the detector; (**b**) dual-channel pyroelectric detector, left filter is reference channel (3.90 μm) and right filter is gas channel (6.25 μm).

To enhance the absorption in a 2–10 μm infrared wavelength, the absorbing layer was covered on the top LiTaO_3_ (LT) crystal surface by the screen printing method [26,27,28,29]. The pyroelectric region included an infrared carbon black absorbing layer (<5 μm)/top electrode layer (Al)/LT crystal (1 mm × 1.8 mm, thickness 20 μm)/ bottom electrode layer (Au). The pyroelectric electrode layer was deposited using a sputtering process with a thickness of 200 nm. The thickness of the metal electrode was measured by a step profiler. Figure 7 (a) shows the carbon black absorption layer at the wafer level prepared on a 3 inch LT substrate. The typical morphology of the carbon black absorption layer is also characterized by the means of scanning electron microscope in Figure 7b. It shows a homogeneous and flat surface. The thickness of the carbon black absorption layer and particle size of the carbon are characterized by scanning electron microscopy in Figure 7c. The thickness of the carbon black absorption layer was 1.95 μm. The particle size of the carbon black absorption layer was 50 nm. Figure 8 shows the proposed dual-channel current-mode circuit diagram. The current mode detector had the advantages of a fast response speed and adjustable gain. In this paper, an optical bandpass filter centered at wavelength of 6.25 μm was used for the pyroelectric detector. Figure 3b shows the absorption spectrum of NO_2_ gas and transmission spectrum of the narrow band-pass filter. The function of the reference channel (3.90 μm) is to reduce the interference caused by the degradation of light sources and water vapor. The dual-channel detector was prepared according to the following steps: (1) Preparation of ceramic circuit board: ceramic circuit board was arranged with resistance, capacitance, and operational amplifiers. (2) Connection of LT: first, the LT was bonded to the ceramic conductive structure with low-temperature silver paste; second, the top electrodes of the LT were connected with the welding pad on the ceramic circuit board using ultrasonic gold wire pressure welding technology. (3) Packaging: dual-channel detectors were welded with TO39 in a N_2_ environment. Figure 9a shows a schematic diagram of the detector. Figure 9b shows the proposed dual-channel pyroelectric detector.

## 4. Results and Discussion

### 4.1. Performance of Pyroelectric Detector

**Figure 10:**
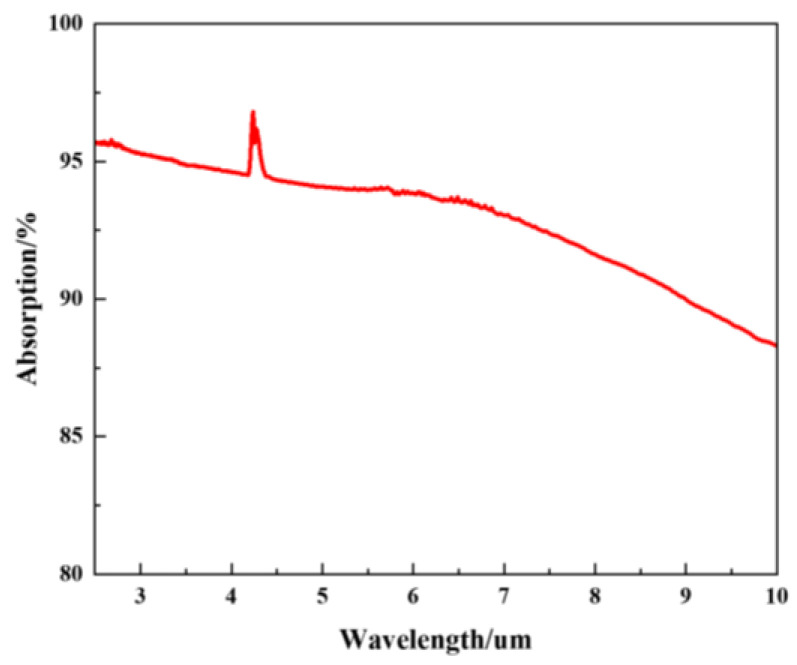
The IR absorption spectrum of carbon black layer.

Fourier transform infrared spectroscopy was used to characterize the properties of the absorbent materials for the pyroelectric detectors developed. The carbon black absorption layer exhibited a broad spectral response. The principle of the pyroelectric detector is that the carbon black absorption layer absorbs infrared light and converts the light signal into a heat signal. Then, the LT layer converts the heat signal into an electrical signal according to the pyroelectric effect. The output voltage of the detector depends on the absorption of the LT pyroelectric detector. A higher absorption of the detector results in a greater output voltage, indicating a greater response. The absorption measurements of the pyroelectric detector over the wavelength range from 2 μm to 10 μm are shown in Figure 10. The absorption of the LT pyroelectric detector was generally >85%. The absorption of detector in the wavelength range of 4.15 μm–4.30 μm ranged from 94–97%. The experimental results show that carbon dioxide in the air can absorb infrared light. Table 2 shows the parameters of the LT pyroelectric detector.

**Figure 11:**
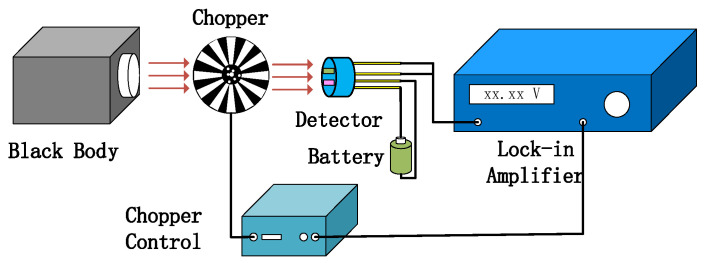
Detector test system diagram.

**Figure 12:**
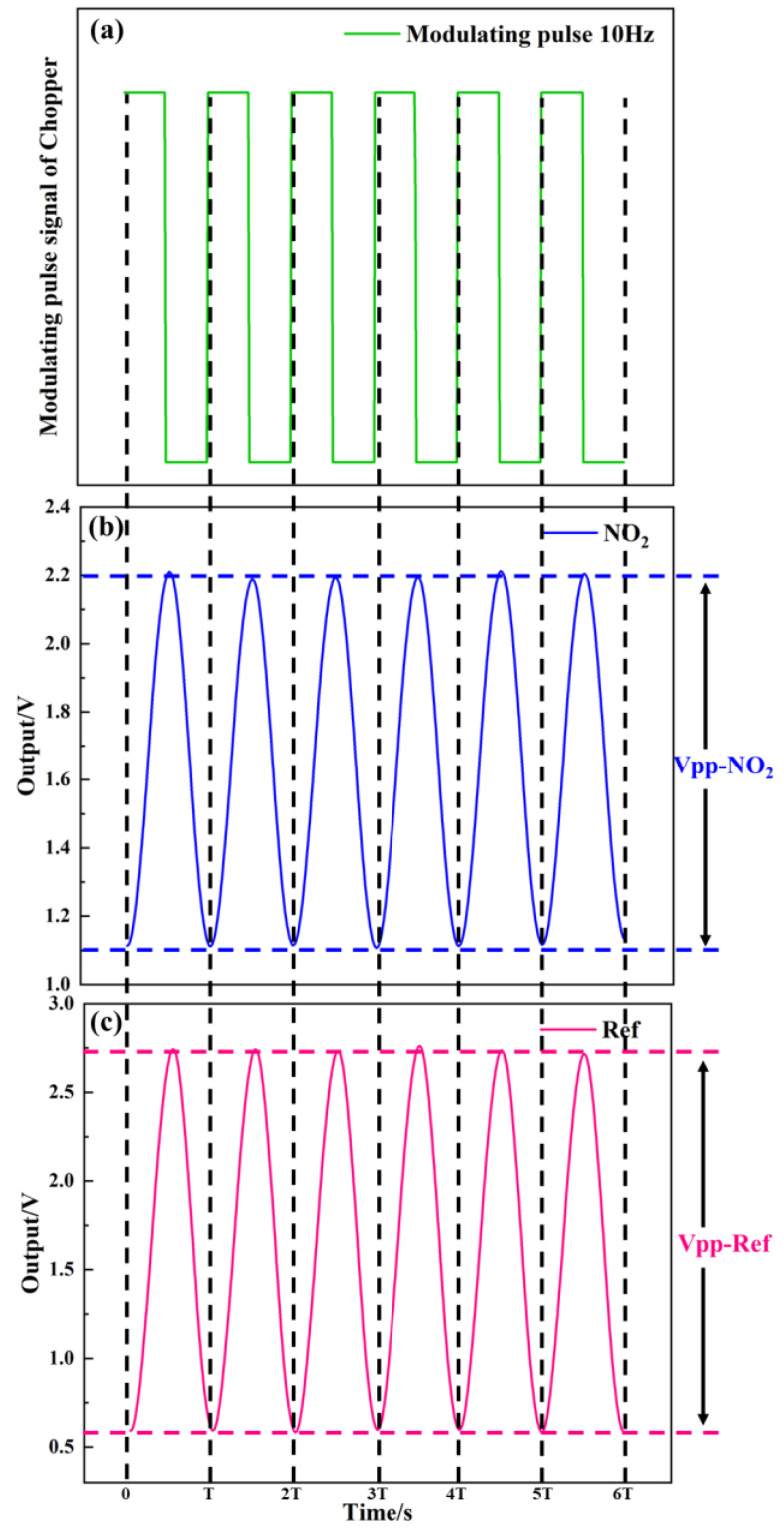
(**a**–**c**) Relationship between modulating pulse, gas channel, and reference channel signals.

As shown in Figure 11, the test system of detector was used to measure the detector output voltage. Figure 12a–c show the relationship between the modulating pulse signals, gas channel, and reference channel. In Figure 12a, the frequency is 10 Hz. Figure 12b–c show the voltage signals of the detector. The voltage of the gas channel was 1.1 V. The voltage of the reference channel was 2.15 V. The voltage responsivity of the detector was calculated to be 1.28 × 10^5^ V/W at a 10 Hz modulation frequency. The noise density of the detector was 40.2 μV/√Hz. The result shows D* 4.19 × 10^8^ cm·√Hz/W.

### 4.2. Calibration and Data Fitting of Gas Sensor

**Figure 13:**
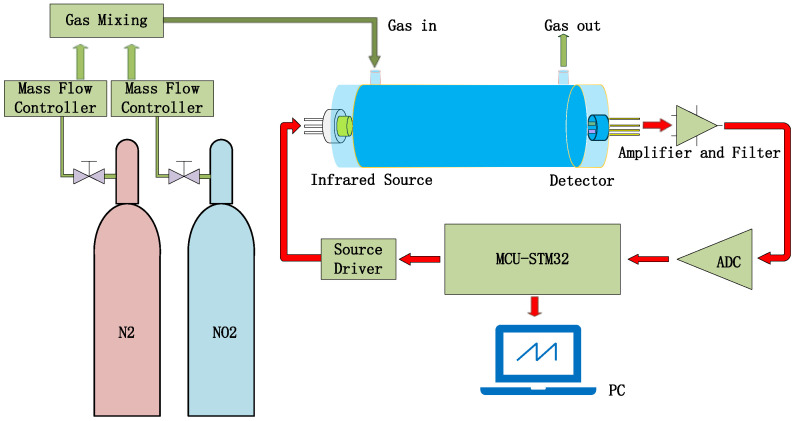
Gas sensor test system diagram.

The testing steps for NO_2_ gas sensing are shown in Figure 13. N_2_ is used as the reference gas and mixed with NO_2_ gas to reduce the concentration of NO_2_ gas [30]. Both gases pass through their respective mass flow controllers before reaching the mixer, which would output the testing gas into the gas-sensing chamber. The output signal of the pyroelectric detector will be collected and processed by the signal-processing circuit.

**Figure 14:**
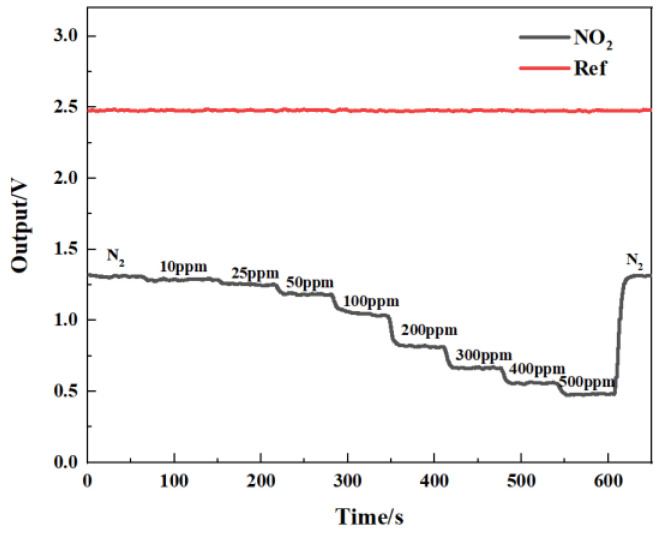
Relationship between gas-sensing voltage response and gas concentration.

**Figure 15:**
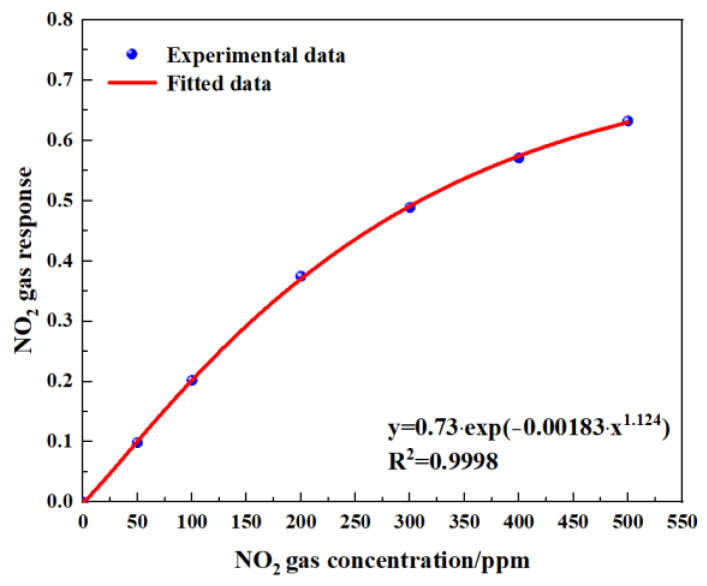
Fitting relationship between gas concentration and gas response.

Figure 14 shows the gas-sensing voltage measured by the detection system at a 10 Hz frequency for an NO_2_ gas concentration ranging from 0 ppm to 500 ppm. The gases were cycled between the reference gas N_2_ and different concentrations of NO_2_ gas. The time duration for each gas to run was set at 1 min. When the gas concentration reached the set value, we measured and recorded the voltage ratio of the two channels. The results showed that in general, a higher voltage response was observed for the long optical cell at a higher gas concentration. The voltage response changed by 900 mV due to 500 ppm NO_2_. Then, the corresponding relationship between the concentration of NO_2_ and the ratio of the voltage was obtained through data fitting. The experimental data of the NO_2_ gas responses in Figure 15 are fitted using the modified Beer–Lambert equation, and we can obtain:

(10) $$y=0.73\cdot exp\left(-0.001183\cdot x^{1.124}\right)$$

where *y* is the fractional absorbance and *x* is the NO_2_ concentration.

**Figure 16:**
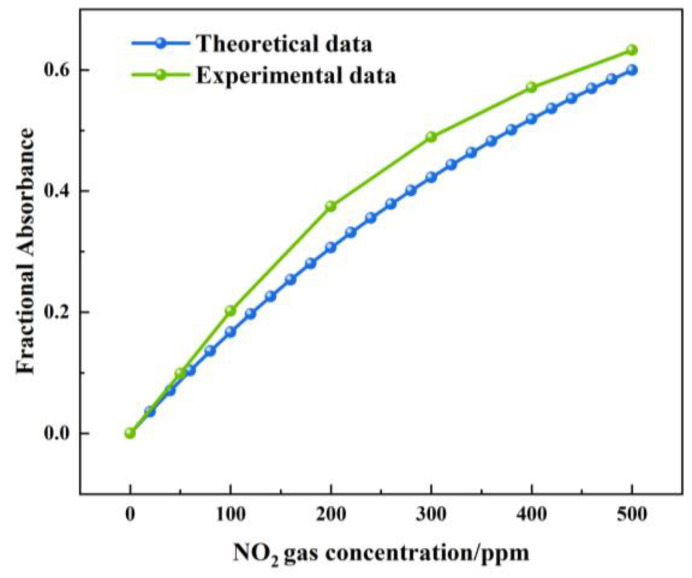
Theoretical and actual values of FA variation with NOx concentration under 1.5 m light path length.

Figure 16 shows the theoretical and actual values of the FA variation with a NOx concentration under a 1.5 m light path length. The theoretical value of the FA variation was between 0 and 0.599. The actual value of the FA variation was between 0 and 0.632. The largest deviation was 0.07, which was observed at a concentration of 200 ppm. The measured value of the FA variation was higher than the theoretical value. The reason was that, when infrared light passed through the gas chamber, a small part of the infrared light was absorbed by the gas chamber.

### 4.3. Relative Error of Gas Sensor

**Figure 17:**
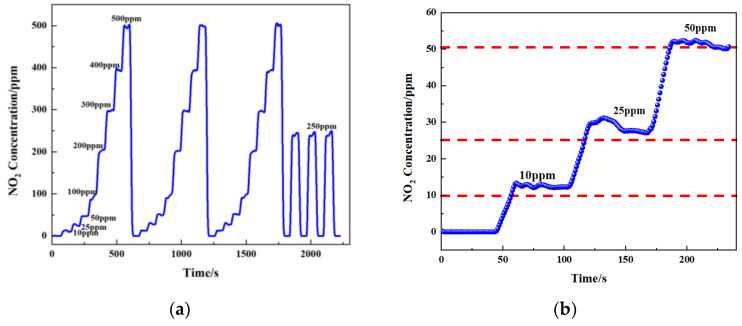
(**a**) Measured concentration of NO_2_ gas response at 0–500 ppm gas concentrations cycled and (**b**) concentration of NO_2_ gas response at 0–50 ppm gas concentrations.

According to the fitted formula, different concentrations of gas were passed in (10 ppm, 25 ppm, 50 ppm, 100 ppm, 200 ppm, 300 ppm, 400 ppm, and 500 ppm), and then the sensor output concentration was detected (Figure 17a,b). To verify the accuracy of the gas sensor, the measured results are shown in Table 3. Tests showed that the maximum error of the sensor was less than 10 ppm in the concentration range of 0–500 ppm. The relative error (δ) was calculated using Equation (11).

(11) $$\delta =\frac{\left(x_{m}-x_{s}\right)}{R}$$

where *x_m_* and *x_s_* are the measurement concentration and true concentration, respectively. R denotes the full scale.

Table 3 shows the NO_2_ concentration between 10 ppm and 500 ppm, and the relative error was between −1.13% and 0.46%. The largest deviation was −1.13%, which was observed at a concentration of 100 ppm, whereas the smallest deviation was 0.26%, which was observed at a concentration of 200 ppm. According to the Beer–Lambert law, monochromatic radiation should ideally be used. When infrared radiation passes through the optical path, the light intensity will change. It will cause differences between the theoretical and actual measurements. The accuracy of the signal-processing circuit also affects the accuracy of the sensor. Therefore, adding a more accurate analog circuit and filter circuit to the signal-processing circuit is helpful to improve the measurement accuracy of the NO_2_ gas sensor.

To calculate the limit of the gas sensor, the measurements obtained seven groups of data. The experimental data of the measured NO_2_ are shown in Table 4. The limit of the gas sensor was calculated using Equation (12):

(12) $$MDL=t_{\left(n-1,0.99\right)}\times S$$

where *MDL* is the detection limit of the gas sensor, *n* is the number of measurements, *t* is the t-distribution, and *S* is the standard deviation of the measured value. When seven groups of data were measured, *t*_(6,0.99)_ was 3.143 [31].

Through the calculation of experimental values, the limit of the gas sensor was 2.8 ppm. The detection limit of the NO_2_ gas sensor depended on the intensity of the light source, the sensitivity of the detector, and the noise of the detector. Although the sensor obtained a low detection limit, it was not the best result. It is necessary to optimize the detection limit of the detector through the data-processing algorithm.

### 4.4. Linearity of Gas Sensor

**Figure 18:**
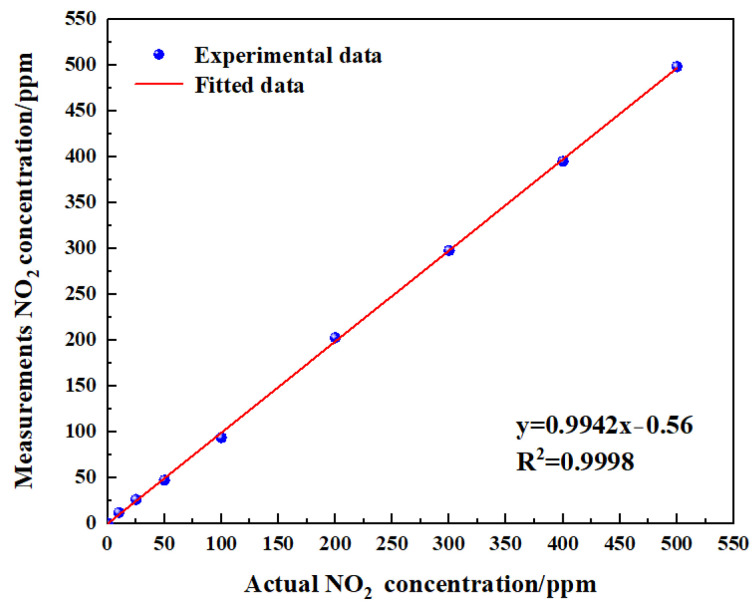
Fitting relationship between actual gas concentration and measurements gas concentration.

To verify the linearity of the gas sensor, the measurements obtained eight groups of data (Figure 18). The experimental data of measurement NO_2_ are shown in Table 5. The linearity was calculated using Equation (13):

(13) $$\delta_{L}=\frac{∆x_{max}}{R}$$

Through the calculation of the experimental and fitting values, the linearity of the gas sensor was −1.09%. It can be concluded from the test results that the gas sensor had a good linearity. Improving the linearity of the sensor was very critical, as reducing the temperature drift of the sensor and increasing the accuracy of signal processing can effectively improve the linearity of the sensor.

### 4.5. Repeatability and Stability of Gas Sensor

To verify the repeatability of the gas sensor, the gas sensor was tested six times, and the experimental data of the measured NO_2_ are shown in Table 6. The repeatability was calculated using Equation (14).

(14) $$S_{r}=\frac{1}{\bar{x}}\sqrt{\frac{\sum_{i=1}^{n}{\left(x_{i}-\bar{x}\right)}^{2}}{n-1}}$$

The long-term stability of the gas sensor was demonstrated inside the chamber. Initially, the chamber was cleaned by N_2_ for 2 min. Subsequently, the concentration of NO_2_ in the chamber were set as 500 ppm. The data interval was set to 24 h, and six data points for each gas were collected. The experimental data of NO_2_ are shown in Table 6. Based on Table 5, the stability *δ_s_* for the multi-gas sensor can be calculated through Equation (15).

(15) $$\delta_{s}=\frac{\left(x_{max}-x_{s}\right)}{R}$$

where *x_max_* and *x_s_* are the maximum drift concentration and true concentration. *R* denotes the full scale.

The repeatability of NO_2_ was 0.47% at a concentration of 500 ppm. The instability of the pyroelectric detector and source lead to a difference in the accuracy of each sampling, which might have led to erroneous measurement results. It can be observed from Table 4 that the stability of the system was 2% at a concentration of 500 ppm. Therefore, the gas sensor had a good repeatability and stability.

It can be seen from the above test results that there were many factors affecting the accuracy, detection limit, and consistency of the NDIR gas sensor. Methods for enhancing sensor performance primarily include improving the performance of pyroelectric detectors, IR emitters, gas chambers, filter algorithm enhancement, and compensation algorithm refinement. To improve sensor accuracy and detection limits, we will focus on enhancing the detector performance and optimizing the signal-filtering algorithm. For pyroelectric infrared detectors, in addition to the design of the sensor structure, the detectivity can be further improved by using thinner LT materials. In addition, thinner infrared-absorbing materials can be used to reduce the heat capacity and further improve the properties of pyroelectric infrared detectors.

Another key measure is implementing a filter algorithm based on FFT to significantly reduce noise levels. The filter’s performance is influenced by the sampling period and MEMS IR emitter modulation frequency. Selecting an appropriate modulation frequency for the MEMS IR emitter ensures the optimal detector performance, while using digital filters based on Fourier transform effectively reduces noise at high frequencies. By combining the MEMS IR emitter modulation frequency with filtering algorithms, we can achieve minimal gas sensor detection limits.

Furthermore, it is important to note that infrared absorbance varies with ambient temperature and humidity conditions. Therefore, we plan to incorporate temperature and humidity compensation algorithms into STM32 to ensure sensor accuracy under varying environmental conditions.

## 5. Conclusions

In summary, a NO_2_ gas detection system based on NDIR spectroscopy was designed and implemented. The system included a developed dual-channel pyroelectric detector with D* value of 4.19 × 10^8^ cm·√Hz/W, a long-light-path white chamber, MEMS IR emitter, and a signal-processing circuit board. Then, an inversion model was established between the output voltage of the detector and the concentration based on experimental data. The gas sensor was capable of detecting concentrations in the range of 0–500 ppm with measurement error of less than 5 ppm for NO_2_. Preliminary experiments indicated that the full-scale relative error of the gas sensor changed by less than 0.46%, with a limit of 2.8 ppm, a linearity of −1.09%, a repeatability of 0.47% at a concentration of 500 ppm, and a stability of 2% at a concentration of 500 ppm. The experimental results demonstrated that the NDIR NO_2_ gas sensor exhibited a good precision, low detection limit, modest stability, and good repeatability. Subsequently, with further improvement of the pyroelectric detector and optimization of the signal-filtering algorithm, the accuracy and detection limit of the developed NDIR NO_2_ gas sensor are expected to be improved, which is expected to be applied in the field of emission monitoring and gas analysis instrumentation.

## Figures and Tables

**Table 1:** The main materials and dimensions of the gas sensor.

Name	Symbol	Values and Units
Materials of detector	/	LiTaO_3_
LT thickness	d	20 μm
MEMS IR emitter power	P	0.66 W
Length of optical path	l	1.5 m
Wavelength of filter	λ	6.25 μm

**Table 2:** LT pyroelectric detector parameters.

Name	Symbol	Values and Units
LT area	A_s_	1 × 1.8 mm^2^
LT thickness	d	20 μm
Absorption coefficient	α	>0.85
Feedback resistance	R_f_	100 GΩ
Feedback capacity	C_f_	0.2 pF

**Table 3:** Calculation of relative error of NO_2_ gas sensor.

Actual Concentration/ppm	Measurements Concentration/ppm			Relative Error (%FS)
10	11	13	13	0.46%
25	27	28	25	0.33%
50	47	48	47	−0.53%
100	92	95	96	−1.13%
200	200	201	203	0.26%
300	298	299	294	−0.60%
400	397	402	395	−0.40%
500	500	497	499	−0.27%

**Table 4:** Calculated detection limit of the gas sensor.

Name	Concentration/ppm							S	MDL/ppm
Experimental data	11	13	13	13	12	11	12	0.899	2.8

**Table 5:** Calculated linearity of the gas sensor.

Name	Concentration/ppm								$∆x_{max}$	Linearity
Experimental data	11.59	25.91	46.96	93.40	202.40	297.42	394.93	498.21	−5.54	−1.09%
Fitted data	9.38	24.30	49.15	98.86	198.28	297.70	397.12	496.54		

**Table 6:** Calculation of repeatability and stability of NO_2_ gas sensors.

Name	Concentration/ppm						Values and Units
Repeatability	503	497	502	499	503	499	0.47%
Stability	501	498	499	496	506	510	2%

## Data Availability

The study did not report any data.
